# Host Directed Therapy Against Infection by Boosting Innate Immunity

**DOI:** 10.3389/fimmu.2020.01209

**Published:** 2020-06-12

**Authors:** Peter Bergman, Rubhana Raqib, Rokeya Sultana Rekha, Birgitta Agerberth, Gudmundur H. Gudmundsson

**Affiliations:** ^1^Division of Clinical Microbiology, Department of Laboratory Medicine, Karolinska Institutet, Stockholm, Sweden; ^2^The Immunodeficiency Unit, Department of Infectious Diseases, Karolinska University Hospital, Stockholm, Sweden; ^3^Infectious Diseases Division, International Centre for Diarrhoeal Disease Research, Bangladesh (icddr,b), Dhaka, Bangladesh; ^4^Biomedical Center, University of Iceland, Reykjavik, Iceland

**Keywords:** phagocytes, gene expression, antimicrobial peptides (AMPs), antibiotic, epithelia

## Abstract

The innate immune system constitutes the first line of defense against invading pathogens, regulating the normal microbiota and contributes to homeostasis. Today we have obtained detailed knowledge on receptors, signaling pathways, and effector molecules of innate immunity. Our research constellation has focused on ways to induce the expression of antimicrobial peptides (AMPs), the production of oxygen species (ROS and NO), and to activate autophagy, during the last two decades. These innate effectors, with different mechanisms of action, constitute a powerful defense armament in phagocytes and in epithelial cells. Innate immunity does not only protect the host from invading pathogens, but also regulates the composition of the microbiota, which is an area of intense research. Notably, some virulent bacteria have the capacity to downregulate innate defenses and can thereby cause invasive disease. Understanding the detailed mechanisms behind pathogen-mediated suppression of innate effectors are currently in progress. This information can be of importance for the development of novel treatments based on counteraction of the downregulation; we have designated this type of treatment as host directed therapy (HDT). The concept to boost innate immunity may be particularly relevant as many pathogens are developing resistance against classical antibiotics. Many pathogens that are resistant to antibiotics are sensitive to the endogenous effectors included in early host defenses, which contain multiple effectors working in cooperation to control infections. Here, we review recent data related to downregulation of AMPs by pathogenic bacteria, induction of innate effector mechanisms, including cytokine-mediated effects, repurposed drugs and the role of antibiotics as direct modulators of host responses. These findings can form a platform for the development of novel treatment strategies against infection and/or inflammation.

## Introduction

After Alexander Fleming first discovered penicillin, several generations of different types of classical antibiotic drugs were developed. Most of these antibiotics target a specific molecule or an essential mechanism needed for survival of the bacterium. However, the targets of antibiotics can be altered by mutations, rendering them ineffective in controlling the infection. Bacterial enzymes can also degrade active antibiotics, and membrane proteins can pump the antibiotic drug out of the bacterial cell and thus prevent it from reaching the intended target. Genes encoding the degrading enzymes and the membrane pumps can be located on plasmids that are horizontally transferred between bacterial strains (1, 2). Excessive antibiotic usage has exerted a selection pressure on the bacterial population, which has increased the spread of antibiotic resistant strains. Although novel antibiotics have been developed the selection of resistance continues, and in recent years, antibiotic resistance has increasingly resulted in prominent problems for healthcare worldwide. Excessive use and abuse of antibiotics are major drivers of antimicrobial resistance (AMR). Livestock and agriculture industries are, for example, major contributors to AMR, due to the application of medically important antibiotics in livestock production and in the food/agriculture industry (3, 4). A critical scenario may emerge with the spread of resistant bacterial strains that no classical antibiotics exhibit activity against, i.e., pan-drug-resistant (PDR) pathogens. Consequently, we are entering an era similar to the pre-antibiotic time, where common infections would be serious or even lethal. Thus, there is an urgent need to find novel strategies to prevent and control infection.

Various alternative strategies have been suggested, including blockage of host microbe interactions, inhibition of virulence factors, and the development of bacterial phage therapy (5). All these approaches have potential but have not yet resulted in therapies that can be utilized in a clinical setting. Furthermore, any single target strategy would theoretically select for resistant bacteria, since novel resistance mechanisms are likely to occur upon application of a new treatment. The consequences of resistance to treatments could be disastrous and thus, the application of new or adapted treatments should be critically studied before introduction into clinical settings.

**Figure 1:**
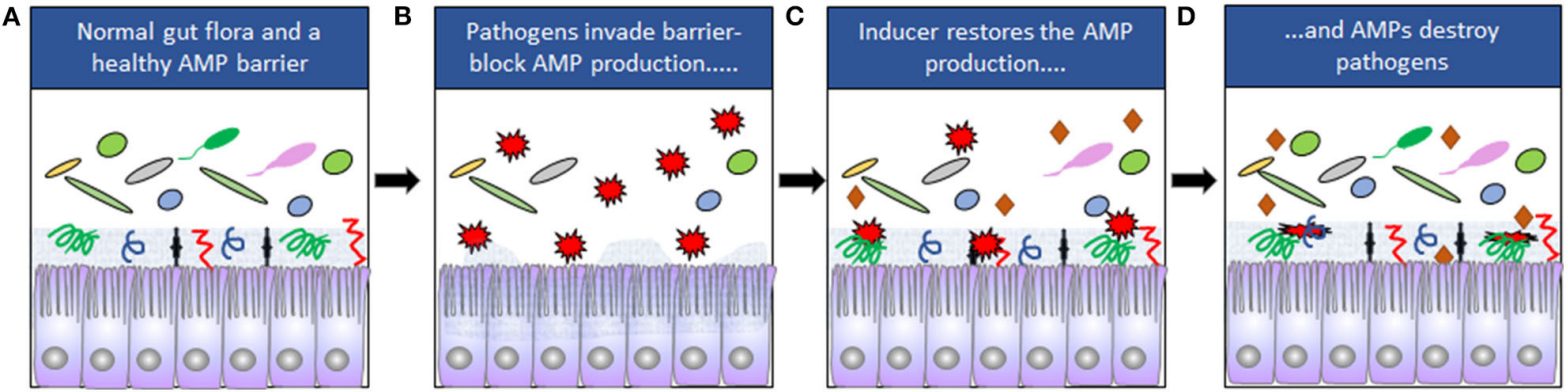
**(A)** The composition of the normal gut microbiota is dependent on expression of AMPs. Mucins are indicated by the gray zone at the apical site of the cells and AMPs by symbols (
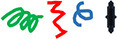
). **(B)** Some pathogens (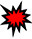) can downregulate the expression of AMPs and disrupt the epithelial barriers. **(C)** Downregulation can be counteracted by inducers (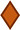). **(D)** The pathogens are eliminated when AMP expression is restored.

We have developed a strategy based on direct stimulation of host cells by activation of endogenous defense mechanisms, such as antimicrobial peptides (AMPs), reactive oxygen species (ROS and NO) and autophagy, in both epithelial cells and macrophages. These are early immune mechanisms aiming to limit the spread of pathogens by killing them [(6–8); Figure 1]. Pathogens have developed different mechanisms to evade the first line of defense and are often not metabolically adapted to a life in the external environment like the lumen of the gut, but instead they need to invade and exploit the host tissues (9). However, counteracting the pathogen-mediated downregulation on host cells by recapturing the expression of innate antimicrobial effectors can lead to elimination of the pathogenic intruder (Figure 1). Interestingly, by this strategy, it is possible to induce multiple genes, generating a powerful response of innate effectors in cells. The genes encoding response factors are co-regulated in an orchestrated fashion to create effective responses and are selected for even in early eukaryotic lifeforms. The simultaneous induction of multiple effectors is the key for this strategy, since the combination should limit the selection of resistant pathogenic strains.

Alternative strategies based on direct application of AMPs have been in development. First, topical treatment using modified peptides on skin infections, relying on direct antimicrobial activity have been presented (10). However, the use of a single peptide would be expected to select for resistant strains. Furthermore, modified AMPs have also been used as injections for immune modulation of the peptides, in order to modify innate responses (11). This second strategy has been reviewed elsewhere (12).

Our approach of using induction of innate immunity with emphasis on the expression of AMPs has resulted in inducers that stimulate a range of innate antimicrobial effectors with limited inflammation. The initial inducers we have studied are butyrate and phenylbutyrate (PBA), which are effective in animal models (6, 13). In human trials, PBA has been used together with vitamin D3 as adjunct therapy to boost immunity against *Mycobacterium tuberculosis* (Mtb) with a beneficial outcome (14). An additional clinical trial utilizing butyrate as adjunct therapy to treat shigellosis showed early reduction of local inflammation (15). Our aim is to develop inducers with optimal properties to activate innate effector mechanisms as host directed therapies (HDTs) to combat infection in the absence of the selection of resistant strains. In addition, a combination of HDTs and classical antibiotics might enhance the treatment efficacy and shorten the duration of antibiotic usage. Indeed, cooperative action between classical antibiotics and innate antimicrobial effectors has been confirmed *in vitro* (16). These combination therapies could retrieve usage of early generations of classical antibiotics and would be in line with improved stewardship, aiming to limit the spread of resistant strains.

Our focus in this review will be induction of antimicrobial effector mechanisms in mucosal epithelial cells and phagocytes of the macrophage lineage.

## Innate Immunity and Front Line Defenses

Innate immunity constitutes the first line of defense and includes specific cells that produce various effector molecules to activate mechanisms resulting in the elimination of pathogens. Different cells of hematopoietic origin constitute the effector cells of innate immunity, such as NK cells and dendritic cells, as well as the professional phagocytic cells monocytes/macrophages and granulocytes. Furthermore, the epithelial cells, while of non- hematopoietic origin, are of fundamental importance, making up a vital surface layer and working as a continuous defense barrier. In our models, the target cells of innate induction have been epithelial cells and macrophages (6, 14). Epithelial cells are sewed and tilted together with tight junctions and adherent junctions, respectively. These linkages between epithelial cells have organ specific adaptations depending on the function of the tissues, such as uptake of nutrients in the small intestine, gas exchange in the lung, and filtration of the blood in the kidney. Moreover, controlled para-cellular transport can also occur. In innate immunity, epithelial cells are functional players and constitutively secrete innate antimicrobial effectors, keeping microbes at bay. Epithelial cells signal to internal sites, secrete specific cytokines, and contribute to defense in the local environment. If microbes pass the epithelial barrier, macrophages, and dendritic cells in the underlying tissue serve as defense mediators with links to adaptive immunity. Further recruitment of neutrophils and monocytes represent another wave of active antimicrobial defenses, originating from the circulation. Innate lymphoid cells (ILCs) are essential in this context, especially in the gut. They sense immunological mediators that are released from epithelial cells and secrete the specific cytokines IL-22 and IL-17 that in turn enhance the secretion of epithelial antimicrobial peptides (17). Indeed, the ILCs have been indicted as important local orchestrators of environmental signals to an immune response for the maintenance of homeostasis. This underlines that the initial defenses rely on a complex network of cell communication (18).

AMPs and reactive radicals, such as nitric oxide (NO) and oxygen species (ROS) with the capacity to eliminate invading pathogens are some of the effector molecules produced by epithelial cells and phagocytes. The combination of these effector systems characterized best in phagocytes, and a similar system operates on epithelial surfaces. Together these effectors work in cooperation in either an additive or a synergistic manner as an efficient armament against microbes. Notably, these effector molecules are evolutionary ancient and have co-evolved with the natural microbiota and have done so without selecting for invasive resistant bacterial strains. This is in contrast to the situation with classical antibiotics that have been only been used for several decades and their usage has selected multiple resistant strains.

Innate effectors, such as AMPs, are not only included in the armament for killing pathogenic intruders but work also as modulators of cell activity. In this respect, AMPs resemble cytokines and chemokines by activating and recruiting different immune cells. Interestingly, this dual activity has been described for several AMPs, indicating a common character. Some AMPs constitute links to adaptive immunity for specific responses and memory functions. As an example, the human cathelicidin LL-37 is chemotactic to human T cells (19), and human beta-defensins can recruit immature dendritic cells and memory T cells (20). Molecules of the adaptive arm of immunity are also included in front line defenses, such as IgA antibodies, that are secreted into the mucosa and make up an efficient barrier together with innate effector molecules. This underlines the cooperation between adaptive and innate immunity at epithelial mucosal surfaces, including multiple effectors for efficient protection of the tissues.

It is of importance to consider the turnover and differentiation of epithelial cells and phagocytes, since the cells have to reach maturity for full activity. Epithelial cells are derived from basal cells in the lungs (21) and from crypt stem cells in the small intestine (22). Upon maturation, epithelial cells use tight, and adherent junction proteins for linking together adjacent cells and seal off the interior tissues from the outside, allowing only small molecules to pass in specific situations. Therefore, tight junctions define a physical character of the epithelial barrier that is important for innate defenses. Microbes do not pass the para-cellular space, with the exception of certain pathogens that actively break the barrier to enter (20, 23, 24). Epithelial cells are exposed to friction and stress and hence are shed continuously and must be renewed. The timing of this is dependent on the specific epithelia. In the small intestine, epithelial cells have a lifespan of ~4 days and in the lung, they last for over 4 weeks (25, 26). The removal of infected cells and renewal of the epithelial cells will maintain active innate defenses. The high turnover of epithelial cells is thus vital for an efficient barrier protection.

Neutrophils are potent phagocytes loaded with a dazzling array of antimicrobial effector molecules. These cells, of hematopoietic origin, differentiate from the bone marrow and enter the circulation as mature phagocytes. The lifetime of the neutrophils is short in the circulation. If they get alarmed through chemotaxis by bacterial compounds, such as N-formyl-MetLeuPhe (fMLF), they will transmigrate from the blood to the site of infection loaded with effectors and exert their functions by phagocytosis of microbes or by release of antimicrobial molecules. It should be mentioned that mouse neutrophilic granulocytes do not produce alpha-defensins, thus questioning results from mouse models as responses in human with reference to studies of these AMPs (27).

As indicated above, mature epithelial cells and phagocytes are constantly alert and ready to kill microbes by constitutive expression of genes encoding antimicrobial effectors. However, both epithelial cells and phagocytes must be activated to enhance the expression of innate immune effectors. Several pathogens suppress these immune effectors as part of their virulence in order to invade the body.

## Downregulation of AMP Expression

The gut microbiota plays a major role in the maintenance of the overall health of the host. AMPs and additional innate effector molecules are generally produced by epithelial cells as well as by circulating inflammatory cells. The epithelial-derived AMP-based defense system has co-evolved with diverse microorganisms and has been shown to regulate the composition of the commensal microbiota (28, 29). Expression of AMPs in the gastrointestinal tract is considered constitutive and has a symbiotic relation to the microbiota. It appears that the natural microbiota is partially spared from the lethal action of AMPs and certain bacteria have adapted resistance. One potential mechanism by which the commensal microbiota reduce their susceptibility to AMPs is by membrane modification, reducing the net negative charge of the bacterial surface, thus decreasing the interactions with AMPs (30). The unique microbiome composition of an individual host is influenced by multiple factors, including the use of specific drugs and antibiotics. Interestingly, activation of endogenous AMPs can eliminate pathogenic bacteria and promote a balanced microbiota in the gut, while conventional antibiotics non-selectively decrease bacterial numbers and diversity in the gut (31).

Different bacterial species thrive in the luminal milieu of the intestinal tract and the microbiota of the gut has evolved by metabolic adaptations. The microbial ecosystems produce a spectrum of metabolites within the human host with diverse functions. Many of these metabolites with specific functions have been characterized and have the ability to limit the growth of surrounding bacteria. The natural microbiota lacks virulence factors needed to break through the epithelium (9). In addition, there is interspecies competition and regulation of the composition between the different bacteria of the natural microbiota. Interestingly, the expression of surface defense molecules is regulated by the microbiota that secrete metabolic products, such as butyrate and lithocholic acid (LCA) (32). Bacterial products modulate the expression of defense molecules that in turn regulate the composition of the microbiota. Butyrate, a short chain fatty acid (SCFA), is produced by fermentation of starches, dietary fiber, sugars, and glycosylated proteins by the intestinal microbiota.

Pathogens exhibit different metabolic adaptation compared to the natural microbiota, and several pathogens have evolved strategies, making them resistant to AMPs [reviewed in Duperthuy (33)]. These strategies include modifications of lipopolysaccharide, modifications of phospholipids, efflux pumps and proteolytic degradation of AMPs. Interestingly, some pathogens in contact with AMPs at low concentration can induce resistant mechanisms and major virulence factors, which would promote their invasive potential.

Furthermore, many pathogenic bacteria have evolved mechanisms to invade the tissue by sophisticated mechanisms to break through the mucosal epithelial barrier in order to avoid or circumvent the defense pathways. We first reported down-regulation of LL-37 and human beta-defensin-1 (HBD-1) expression in human biopsies of patients with early stage of *Shigella* infection (34). Plasmid DNA appeared to be responsible for turning off the expression of these AMPs in the epithelial surface as a virulence strategy to escape the immune surveillance. Others also confirmed the downregulation of AMPs, and secreted proteins were claimed to be the mediators responsible for this downregulation (35). As a proof-of-principle, using an animal model of shigellosis, we were able to demonstrate that *Shigella* spp. down-regulated CAP-18 (rabbit cathelicidin) in the large intestine, at the site of infection (13). Interestingly, downregulation of CAP-18 was also observed in remote epithelial lining of lungs and trachea, where no infection occurred (6). Later, it was shown that *Vibrio cholerae* and enterotoxigenic *Escherichia coli* (ETEC) suppress LL-37 and HBD-1 expression in intestinal epithelial cells through the actions of cholera toxin (CT) and labile toxin (LT), respectively (36). A similar scenario was observed in patients with cholera and ETEC diarrhea during early infection (37). Furthermore, our group showed that pathogenic strains of *Neisseria gonorrhoeae* downregulated the expression of LL-37 in a human cervical epithelial cell line, while non-pathogenic or heat killed pathogenic strains of *Neisseria* did not have such an effect. This result suggested a survival strategy of the pathogen during invasion of the genital tract (38). In experimental studies, we further demonstrated that *Vibrio cholera* or enteropathogenic *E. coli* (EPEC) also can downregulate CAP-18 in the small intestinal epithelia (39, 40).

These studies led to the conclusion that counteracting or blocking pathogen-mediated down-regulation of endogenous AMPs could be used for treatment of infections. Various small molecular compounds are known to induce endogenous AMPs, where immunomodulatory properties could be harnessed to treat infections. Host directed therapy (HDT) against pathogens by boosting the expression of endogenous AMPs is thus considered as a novel alternative for treatment of infectious diseases (Figure 1).

## Induction of Innate Effectors in Phagocytes and Epithelium

AMPs are potent antibacterial substances with activity comparable to classical antibiotics. Initially it was suggested that the peptides could be utilized in therapy as direct antimicrobial substances. The disadvantage with this approach is that the peptides are enzymatically degraded in the gastrointestinal tract, and when injected they might evoke production of antibodies, limiting the usage to topical administration for skin infections and treatment of wounds. Furthermore, monotherapy with single synthetic peptides could lead to selection of resistant strains (41). Therefore, our logical approach was to stimulate AMP production by activating regulatory pathways in the cells, without stimulating inflammatory responses through NFκB. This would result in induction of several genes encoding active peptides, mimicking the true response in our front-line defenses (42). Accordingly, small molecules that can be administrated orally have been used as inducers of AMP-expression both *in vitro* and *in vivo*. By this strategy multiple peptides with different killing mechanisms together with NO and ROS would be engaged, making selection of resistant bacterial strains unlikely. These small inducing molecules can enter the blood stream and have systemic effects, as well as also boosting innate defenses at mucosal surfaces and in phagocytic cells (6, 43–46).

The idea to improve mucosal defenses was proposed by Fehlbaum et al. (47) where they showed that isoleucine could induce transcription of human beta defensin 2 in bovine epithelial cells. Shortly after this, we identified butyrate as a major inducer of AMP-expression (48, 49). Butyrate is a product of fiber fermentation by the natural microbiota and the carbon energy source for colonic epithelial cells. Butyrate is known to be an HDAC inhibitor and has earlier been used in a *Shigella* infection model with beneficial effects (50). However, the mechanisms underlying these beneficial effects remained elusive at that time (51).

Today several of the inducers of AMP expression have been identified as histone deacetylase inhibitors (HDACi) (52). The activity relates to histone modifications, where histone acetyltransferase (HAT) mediates acetylation of lysine residues, resulting in reduced ionic interactions between the basic histones and the acidic DNA with more open chromatin structure. In contrast, HDAC reverses this reaction, and if inhibited the histones are maintained in the acetylated form. Therefore, HDAC inhibition affect the chromatin structure from tightly packed toward open with enhanced access of different transcription factors and machinery for transcription, including the RNA polymerase II. Typically, the responses to HDACi are complex and involve many genes, and some pathways of HDACi are physiologically relevant. Butyrate is considered an important part of the normal human physiology together with other short chain fatty acids (SCFA), enhancing the expression of mucins and AMPs in intestinal epithelial cells. SCFAs strengthen defenses and enhance epithelial functions by increasing tight junctions and thus maintaining barrier integrity. Various bacterial species produce butyrate in the human colon, most of them belong to the Firmicutes phylum, in particular the clostridial clusters IV and XIVa, which have been associated with a healthy gut homeostasis (53). Altered composition of the microbiota with reduced growth of butyrogenic *Clostridium* species has been linked to susceptibility to infections and inflammation (54).

Butyrate also affects various immune cells in the gut with beneficial effects of the host, balancing inflammatory reactions to homeostasis of host microbe interactions in the gut (55). The specific receptors of butyrate GPR41 (FFAR3) and GPR43 (FFA2) are G-protein coupled receptors that have been characterized on several immune cells (56). The anti-inflammatory properties of butyrate have been shown to be dependent on the expression of GPR43 in regulatory T-cells (57). Butyrate can also enter cells for example via the SLC5A8 transporter (58), which is needed for the HDACi activity. In summary, both the HDACi and receptor activation by butyrate are needed for imparting the beneficial effects of butyrate, leading to enhanced barrier integrity (59).

The concentration of butyrate in the gut is 2.3–26.1 mmol/kg, while it is 1–64 μM (60) in the blood stream. Accordingly, butyrate can be absorbed from the colon, and affect remote organs, which we have shown for the pancreas and the lungs (6, 44).

Butyrate is a foul-smelling compound and is not possible to use it as an oral drug. Therefore, we searched for alternative compounds and identified phenyl-butyrate (PBA), a drug already approved for treatment of urea cycle disorders by virtue of the ammonium scavenging capacity (61). Importantly, PBA is available in tablet form and using animal models, we have confirmed that it has a similar induction profile of AMPs as butyrate (6). It is also an HDACi as well as a chemical chaperone (62). Since PBA is a registered drug for clinical use, all toxicity, and regulatory studies have already been performed and thus, it could enter the clinic in a fast track for additional indications (Phase IIB-trials).

The most recent research on butyrate further underlines its important role. In a mouse model, butyrate was found to imprint antimicrobial programs in macrophages (63). Butyrate was demonstrated to increase antimicrobial activity, reduce mTOR kinase activity, increase LC3-associated host defense, as well as enhancing AMP expression without increasing the production of inflammatory cytokines. The antimicrobial response was dependent on HDAC3-expression, which was shown by blocking specific transcription using siRNA (63). In line with this result, butyrate was shown to protect intestinal epithelial cells from damage caused by *Clostridium difficile* in a mouse model (64). This effect was mediated by stabilization of the transcription factor HIF-1α, resulting in attenuated inflammation and improved intestinal barrier. Thus, butyrate is a key player in host defense against inflammation and infections (64). The pathway involving HIF1α has also been shown to be important in the inhibition of *Candida albicans* by commensal bacteria, where cathelicidin was highlighted as an important contributor (65).

HDAC inhibition has long been known and, as the name indicates, relates originally to the effect on histones and chromatin structure. In recent years, acetylation has been shown to be much broader, comprising many non-histone proteins. These proteins are involved in key cellular events including signal transduction, autophagy, metabolism, protein folding, and cell division (66). Accordingly, the acetylation was renamed to lysine acetyltransferase (KAT) and lysine deacetylase (KDAC) to underline broader targets. These epigenetic mechanisms have recently been thoroughly reviewed in relation to innate immunity and infections (67). Clearly, the regulatory circuits of HDACi, or rather KDACi, in relation to induction of AMPs are complex and many specific details are still to be clarified. Therapeutic interventions enhancing defenses by affecting these regulatory pathways to prevent or fight infections are thus worth pursuing. Adverse effects have been of concern for HDACi and must be considered for each inducing compounds. However, HDACi is a part of our physiology exemplified by the production and release of butyrate in the gut.

We have shown co-operative inducing activity between butyrate/PBA and vitamin D3 on the expression of the *CAMP* gene encoding LL-37 in lung and gut epithelium (68). The cooperativity with respect to the *CAMP* gene relates most likely to effects on different regions of the promoter. Interestingly, in lung epithelial cells with the VDR expression knocked-down by siRNA, the induction of the *CAMP* gene by PBA and butyrate was reduced (69). Butyrate and PBA likely altered chromatin structure and thus increased the access of VDR to the *CAMP* gene promoter. PBA and butyrate are not cognate ligands to VDR, but acetylation of components in the signal transduction pathway of VDR is the most likely explanation for the observed cooperative effect.

Vitamin D3, a nutritional component, has been shown to be a potent AMP inducer. This activity of vitamin D3 was originally found in a general screen of vitamin D3 induced genes. The induction of the *CAMP* gene expression was outstanding compared to other affected genes identified (70). Subsequently, the effects of vitamin D3 on AMP-expression in relation to tuberculosis (TB) and leprosy were demonstrated both *in vitro* and *in vivo* (71), and the anti-mycobacterial activity was dependent on the *CAMP* gene (72). Binding of the vitamin D receptor (VDR) to the promoter of the *CAMP* gene, with subsequent production of the human cathelicidin LL-37, was shown by Gombart et al. (73) and more specifically in the skin by Weber et al. (74). Importantly, the vitamin D3 effect was specific for cathelicidin-expression in primates and was dependent on a transposable element that entered the gene late in evolution (75). Rodents do not have this insertion element and hence the mouse has not been a suitable *in vivo* model for vitamin D-mediated responses against infections. However, a mouse model of the vitamin D-induced human cathelicidin was recently generated. This transgenic mouse has some of the features seen in humans after vitamin D3 induction, with enhanced killing of *Staphylococcus aureus*, as well as induced *CAMP* gene expression following topical induction in the skin (76).

Notably, vitamin D3 has been known to induce the differentiation of monocytes into macrophages. The presence of vitamin D during differentiation promotes the expression of cathelicidin and intracellular control of mycobacterial growth (77, 78).

The observation of the synergy between PBA and vitamin D, together with the potent effect of *CAMP* expression in the lung epithelial cell-line VA10, and against Mtb prompted us to continue with clinical studies. Previous clinical trials using vitamin D as an adjunctive therapy together with standard TB-treatment have failed to show positive effects on pre-specified clinically relevant endpoints (79). However, a significant effect on time to sputum culture conversion was observed when a polymorphic variant (TT genotype) of the VDR receptor variant TaqI was present (80).

Interestingly, in our study using PBA and vitamin D as adjunct therapy together with the four classical TB drugs Isoniazid, Rifampicin, Ethambutol, and Pyrazinamide, enhanced the antibiotic activity. Furthermore, the outcome was significant for sputum clearance together with a decline in clinical score. These results indicate that host directed therapy is effective against difficult to treat infections (14).

Additional inducers would be of interest to broaden our concept for host directed therapy. We therefore set up a strategy to screen for novel inducers that included the development of a reporter-system cell line containing the *CAMP* gene fused to luciferase (52). The initial screen was with a panel of HDAC inhibitors followed by a Prestwick library of 1,200 compounds of FDA approved drugs. Several compounds were identified as *CAMP* gene inducers, but the HDACi Entinostat gave the most prominently induced response at a low concentration (2.5 μM). Subsequently, we showed Entinostat to be effective in animal models for *Shigella* and *Vibrio cholera* infections (8, 40). Detailed analyses of the mechanism for induction of Entinostat was dependent on the transcription factors STAT3 and HIF1α. HIF1α was found bound to the *CAMP* gene promoter, while STAT3 activates HIF1α (81), but the initial steps of the activations are still not clarified. Since Entinostat is toxic at high concentrations and has been developed as a cancer drug candidate, we therefore made several variants of Entinostat called aroylated phenylenediamines (APDs), with the aim to improve water solubility and to reduce toxicity (8). Several of these non-toxic lead compounds are active in inducing several AMP-genes in colon and lung epithelial cells (8, 82). We are currently in the process of developing these compounds further as drugs for host directed therapy against infection. The approach is to utilize these novel inducers alone or in combination with conventional antibiotics.

The effects of vitamin D on the *CAMP* gene are well-documented and relevant for *Mycobacterium* infections, both leprosy and tuberculosis (71, 78). The vitamin D receptor (VDR) binds to DNA as a heterodimer in complex with the retinoid X receptor (RXR). The role of the RXR partner in this context has not been defined in detail. Partners of class II nuclear receptors, other than VDR, are farnesoid X receptor (FXR) and the retinoic acid receptor (RAR). Interestingly, these partners are also linked to AMP induction and innate immunity.

Bile acids are ligands to FXR that can induce *CAMP* gene expression. The *CAMP* gene induction by FXR was initially linked to biliary duct sterility (83). In mouse models, utilizing knockout mice of the *CYP27* gene with reduced bile synthesis or knockout mice for the *FXR* gene (NR1H4 gene), a broader effect of FXR was seen to regulate innate immunity in small intestinal epithelial cells (84). FXR and bile acids regulated the expression of several antimicrobial components and affected the occludin protein of the tight junctions (84). Pronounced effects of deleted FXR or reduced bile synthesis on antimicrobial activity and bacterial translocation were observed. FXR has also been highlighted in inflammation as counteracting TLR-4 mediated response in myeloid cells (85).

Furthermore, the synthesis of retinoic acid, the ligand for RAR/RXR heterodimer, has been linked to epithelial transcription programs of defense. Commensal *Clostridia* species can modulate retinoic acid availability by affecting the synthesis and thereby the IL-22-dependent antimicrobial responses. Bacterial regulation of retinoic acid synthesis was shown to be important for the balance of the microbiota (86), indicating a role for RAR/RXR in the homeostasis of the natural microbiota.

Other orphan members of nuclear receptors with unknown natural ligands have emerged as crucial regulators of inflammation and immune responses (87), but are not directly linked to induced expression of AMPs.

**Figure 2:**
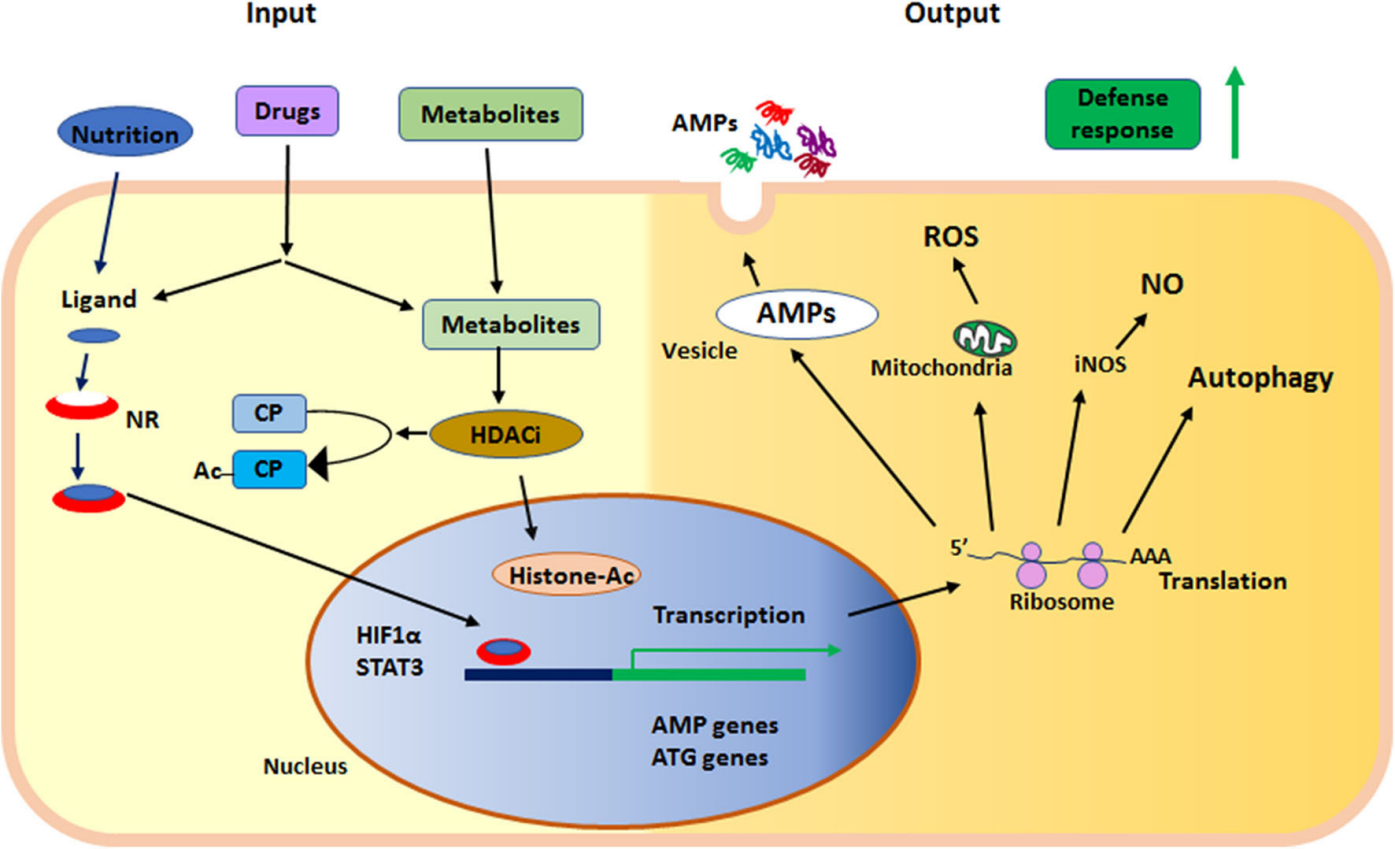
Summary of the main events in early defense by innate effector mechanisms described in the text. NR, nuclear receptor; HDACi, histone deacetylase inhibition; AMPs, anti-microbial peptides; ROS, reactive oxygen species; NO, nitric oxide; iNOS, inducible nitric oxide synthase; ATG, autophagy related gene; HIF1α, hypoxia-inducible factor 1-alpha; STAT3, signal transducer and activator of transcription 3; CP, cytoplasmic protein; Ac, acetylation; Histone-Ac, acetylated histone and open chromatin.

In summary, nuclear receptors and KDACs are sensors of metabolites and nutritional components, regulating the status of innate immunity. Together these proteins serve as sensors in the epithelium at the interphase of the outside environment and the tissue, where microbial exposure occurs. Both nuclear receptors and KDACs can be affected by synthetic ligands and are thus motives for enhancing defenses to prevent or fight infections (Figure 2).

## Activation of Autophagy to Fight Bacterial Infection

Autophagy is a conserved mechanism to maintain homeostasis and is tightly linked to cellular metabolism. It is also involved in the cellular defense mechanisms against a range of intracellular microbes. Several important primary pathogens, including Mtb, *Salmonella*, and *Legionella*, have evolved mechanisms to avoid or block the autophagic process (88). Thus, efforts to restore autophagy by using small molecular drugs could be used to fight infections against these bacteria. Here we will review recent reports and results, including drugs that have been used for this particular purpose.

Autophagy is a general concept that can be further subdivided into macro-autophagy, mitophagy, xenophagy, LC3-associated phagocytosis, and chaperone-mediated autophagy, with specific but also overlapping functions (89). Here we will mainly discuss xenophagy, which specifically targets bacteria (90).

Mtb specifically targets autophagy and blocks the fusion between the phagosome and the lysosome (91, 92). This mechanism facilitates the intracellular survival of Mtb in human macrophages and is essential for the virulence of Mtb. Thus, restoration of autophagy has been selected as a target for host-directed therapies against Mtb. Several FDA-approved drugs with the potential to restore autophagic function during Mtb-infection have been identified. Vitamin D, for example, has been known to have beneficial effects against Mtb since the pre-antibiotic time, but the mechanisms have not been well-described. However, Liu et al. (71) showed that intracellular control of Mtb growth is regulated by vitamin D via the induction of the AMP LL-37. LL-37 has direct anti-mycobacterial effects *in vitro*, but other mechanisms may also be involved. One intriguing possibility is that LL-37 acts as a mediator of autophagy activation (93), which is a claim that has been further substantiated by our results, showing that vitamin D activates autophagy via a paracrine loop and activation of the surface associated P2X7-receptor (7). In fact, the autophagy-related genes Atg5 and Beclin-1 are induced by vitamin D in human macrophages (7, 93). It was recently shown in a *Helicobacter pylori*-model that vitamin D can restore lysosomal degradation by activation of the protein disulfide isomerase family A, member 3 (PDIA3) receptor via upregulation of Ca^++^ channels, resulting in a normalized lysosomal acidification (94).

Notably, the autophagy-activating effect of vitamin D is further enhanced by the addition of PBA and the combined effect of these compounds depends on LL-37 in order to activate autophagy. The mechanism behind this effect involves VDR-activation but most likely also chromatin remodeling caused by the histone deacetylase effects exerted by PBA (7). In addition, PBA alone has a direct growth inhibitory effect on Mtb (95). Due to the promising effects of vitamin D on autophagy and inhibition of Mtb growth in human macrophages, several clinical trials of vitamin D supplementation as adjunctive treatment to patients with pulmonary TB have been performed (79). However, the clinical outcome in these trials have been limited in relation to the primary endpoint (sputum culture conversion). The combination of vitamin D with PBA resulted in a faster conversion of sputum culture and better clinical TB-score in two clinical trials (14, 96). Furthermore, some positive effects of vitamin D3 have been observed with regard to pre-selected secondary endpoints, including pro-inflammatory cytokines (97).

The discrepancy between the *in vitro* results showing beneficial effects of vitamin D3 and the mostly negative results from clinical trials is intriguing. One reason for the lack of effect of vitamin D3 in clinical TB-trials is that the standard treatment is usually very effective, and any beneficial effect of adjunctive vitamin D3 treatment would require a very large study cohort to detect a statistically significant difference. Based on these assumptions it has been proposed that the true benefit of an adjunctive protocol with any immunomodulatory drug would be found in trials on MDR TB-patients, where the standard drug regimens are ineffective. In fact, a recent meta-analysis comprising 1,850 participants in 8 interventional trials found that vitamin D3 accelerated sputum culture conversion in patients infected with MDR Mtb, but not in those patients with susceptible Mtb isolates (79). Another important factor may be the daily dose vs. bolus dose of vitamin D3 given over a period of weeks or months, which may not be suitable to impart beneficial impacts on the immune system to fight TB disease (14, 96). Finally, vitamin D3 supplementation has been shown to exhibit the best effect against respiratory tract infections in individuals with prominent vitamin D3 deficiency (<25 nmol/L). However, the beneficial effect was evident also in those individuals with serum levels of 25 OH-vitamin D3 up to 75 nmol/L (98).

On the other hand, it has been shown that the common TB-drugs isoniazid and pyrazinamide activate autophagy (99). The beneficial effect of this *in vitro* finding remains to be shown in a clinical context, but adds another layer of complexity to the story.

In addition to vitamin D3, several other drug-like compounds have been identified in different screens for autophagy inducing agents. For example, the anti-parasitic drug nitazoxanide, the anti-depressive drug fluoxetine and the EGFR-inhibitor gefitinib, have all been shown to activate autophagy. In addition, the anti-epileptic drugs carbamazepine and valproic acid also activate autophagy and impair Mtb growth in human macrophages (100).

Autophagy is also important for host defense against other bacteria, not classically considered to have an intracellular lifestyle. One example is *Klebsiella pneumoniae*, where downregulated or impaired autophagy leads to increased bacterial growth and increased mortality in mice (101). However, it should be noted that for some additional extracellular bacteria, such as *Staphylococcus aureus* and *Pseudomonas aeruginosa*, autophagy appears to be exploited by the pathogens to cause disease. Consequently, impaired autophagy has been shown to lead to a beneficial outcome in infectious models (102–104), which represent a reverse situation in comparison to Mtb and other strictly intracellular bacteria.

To conclude, activation of autophagy clearly seems to have beneficial effects during infection with Mtb, but for other bacteria the effects might be different. Thus, it is important to delineate the role of autophagy for specific bacteria since modulation of autophagy could potentially lead to beneficial or detrimental effects, depending on the context.

## Antibiotics and Effects on Host Immunity

Antibiotic treatment is the natural first choice of treatment against bacterial infections. The traditional paradigm considers that antibiotic drugs specifically target bacteria. However, several antibiotic drugs also affect innate immunity via direct effects on host signaling pathways. The most potent antibiotic drug classes in this respect are the quinolones, the macrolides and drugs used for treatment of tuberculosis, mostly studied for the first-line drug rifampicin. In this section, we include examples of antibiotics that have been shown to have direct effects on host immunity.

Ciprofloxacin, one of the best-studied antibiotics of the quinolone class, was developed as a potent inhibitor of topoisomerase 2 in bacteria. It is active against both gram-positive and gram-negative bacteria. It is widely used by virtue of its good and broad intracellular penetration effects and low adverse event profile. However, resistance against ciprofloxacin has emerged, and consequently the recommendations regarding empirical use of this drug have been changed. In addition, potentially serious adverse side effects are also increasingly acknowledged. These side effects include cardiac arrhythmias, tendon ruptures, and the selection for *Clostridium difficile*-associated diarrhea (CDAD).

Ciprofloxacin has a direct bactericidal effect against bacteria via the SOS-response, which releases large amounts of ROS, causing DNA-damage, and bacterial death (105). Consequently, scavengers of ROS inhibit the effects of ciprofloxacin, which provide evidence for a ROS-dependent bacterial killing. It is well-known that ciprofloxacin has profound effects on the intestinal microbiome. In fact, an altered microbiota was evident up to 1 year after treatment of ciprofloxacin (106, 107). Clinical use of ciprofloxacin is associated with a higher risk of developing CDAD, which could possibly be mediated via direct effects on the microbiota (108). However, ciprofloxacin has also been shown to downregulate colonic expression of AMPs (109). Recently, it was also shown that ciprofloxacin caused a loss of the mucosal barrier and immune-function in the intestine (110). Thus, it is becoming increasingly clear that ciprofloxacin has potent and direct effects on innate immunity. For example, ciprofloxacin has been shown to upregulate IL1-β and TNF-α expression in human macrophages (111). In a clinical study, patients with gram-negative sepsis that were treated with ciprofloxacin exhibited lower levels of pro-inflammatory cytokines, compared to those patients treated with a beta-lactam antibiotic (112). To conclude, ciprofloxacin is widely used and it has a profound impact on the microbiota. However, ciprofloxacin also exerts immunomodulatory functions, which may be beneficial (anti-inflammatory) or detrimental (down-regulation of AMP-expression) depending on the circumstances.

Azithromycin (AZM) is a macrolide antibiotic drug targeting the 50S subunit of the bacterial ribosome. It is widely used against respiratory tract infections, since it is also active against atypical bacteria, including *Mycoplasma* spp., atypical mycobacteria and *Legionella* spp. It also has been extensively used as a prophylactic drug in patients with asthma and bronchiectasis. The prophylactic use of AZM was further substantiated recently in a randomized and placebo-controlled trial, where the number of asthma exacerbations was reduced, and the quality of life was increased (113). A Cochrane review has also concluded that there are beneficial effects of AZM in patients with bronchiectasis (114). The drawbacks are the potential risk of the development of resistance against AZM and adverse events, such as arrhythmias, diarrhea, and arthralgia (115).

In addition to its bacterial target, AZM has potent immuno-modulatory effects. These effects are considered to contribute to its efficacy as a preventive agent in patients with respiratory diseases. For example, it was recently shown that AZM protects against *Pseudomonas* infections in the lung via direct inhibition of the inflammasome (116). In addition, AZM polarizes macrophages toward an M2 phenotype via inhibition of STAT1 and NFκB (117). AZM also contributes to the strengthening of the barrier in the airways by induction of tight junction proteins (118). Further, AZM induces epidermal differentiation, which potentially can protect the lungs during infection (119). Another layer of recently added complexity is that AZM was shown to alter the microbiome and metabolome of the lung, promoting bacterial metabolites with anti-inflammatory effects of the airways. Finally, AZM may protect against virus infections, as shown in the context of zika-virus, where AZM induced protective type 1 and type 3 interferon responses (120). The antiviral effects of AZM could also explain the beneficial effects in asthmatic patients, where rhinovirus often triggers exacerbations (121). One way to reduce the problems with antibiotic resistance is to use a macrolide analog without antimicrobial properties but with retained anti-inflammatory effects. One such non-antibiotic macrolide drug candidate was recently shown to reduce inflammation in an allergy mouse model (122).

A third group of antibiotics with potent interactions with the immune system is the TB-drugs. This has been best studied for the first-line drug rifampicin (RIF), which has an impact on the immune responses during TB-infection at several levels. One key mechanism appears to involve direct interactions between RIF and nuclear receptors. For example, it has been shown that RIF activates pregnane X receptor (PXR) that can promote TB growth in macrophages via increased efflux of TB-drugs out of the cell (123). This leads to sustained bacterial growth in the presence of the drug, which may accelerate the development of resistance. Notably, *M. tuberculosis* with a single nucleotide polymorphism in the rpoB-gene bypasses the protective IL1-β response and instead promotes an interferon-β response. This causes metabolic reprogramming of macrophages, ultimately promoting bacterial growth (124). A recent study links the aryl hydrocarbon receptor (AhR) to TB-treatment. AhR is a member of the family of basic helix-loop-helix transcription factors and can be activated by tryptophan metabolites. RIF, and the related drug rifabutin, are sensed by the AhR, leading to impaired phagocytosis of Mtb by macrophages. The application of a small molecule inhibitor of AhR caused decreased phagocytosis and improved killing of Mtb (125). These findings collectively demonstrate that TB-drugs are sensed by the innate immune system, which activate changes in host cells that may be detrimental for the control of intracellular bacterial growth. Nevertheless, the clinical implications of innate immune sensing of TB-drugs remain to be shown. It should be noted that rifampicin has been the most essential drug for TB-treatment for decades. However, it is possible that drug concentrations below the MIC-value will impair endogenous defenses, as suggested by Puyskens et al. (125), which could play a role in the resolution of infection.

## Conclusion

Initial innate defenses at epithelial surfaces are complex and include AMPs as important effectors. Many of the surface effectors including ROS and NO are also present in phagocytes, the second line of early defenses. The expression of AMPs is included in the differentiation program of innate immune cells but is also influenced by signals from the environment. Over the last decade, it has emerged that metabolites from bacteria and nutritional components are influential environmental mediators for the expression of the innate immune effectors. These innate components constitute an important barrier against invading pathogenic microbes, preventing them from entering the internal host environment. The strength of the barrier is decisive for keeping microbes at bay and the environmental signals are important for a tight barrier. Several pathogens suppress the expression of barrier components and thereby manage to open an entrance into the body, leading to infections. Details behind the interactions between bacteria and host cells must be resolved for each pathogen and much work remains to be done. However, powerful analytical models based on gene deletions in bacteria and host cells from different organisms are developing fast and open up for novel treatment approaches. A detailed knowledge on pathogen-mediated suppression of innate immunity and the discovery of counteracting compounds is the focus of our research. For example, inducers of innate effector mechanisms may be used alone or in combination with antibiotics. Such approaches would likely reduce the spread of resistance strains. Future research efforts on the interaction between multidrug-resistant bacteria and the host is warranted. The use of host directed therapy holds the promise to promote health and reduce the spread of antibiotic-resistant strains.

## Author Contributions

PB, RR, BA, and GG planned the outline, wrote, and edited the manuscript. RSR edited and made the figures. All authors contributed to the article and approved the submitted version.

## Conflict of Interest

The authors declare that the research was conducted in the absence of any commercial or financial relationships that could be construed as a potential conflict of interest.
